# Hippocampal subfield volumetry in patients with subcortical vascular mild cognitive impairment

**DOI:** 10.1038/srep20873

**Published:** 2016-02-15

**Authors:** Xinwei Li, Deyu Li, Qiongling Li, Yuxia Li, Kuncheng Li, Shuyu Li, Ying Han

**Affiliations:** 1 Key Laboratory for Biomechanics and Mechanobiology of the Ministry of Education, School of Biological Science & Medical Engineering, Beihang University, Beijing, 100191, China; 2 Center of Alzheimer’s Disease, Beijing Institute for Brain Disorders, Beijing, 100053, China; 3 Department of Neurology, Xuan Wu Hospital, Capital Medical University, Beijing, 100053, China; 4 Department of Neurology, Tangshan Gongren Hospital, Tangshan, 063000, China; 5 Department of Radiology, Xuan Wu Hospital, Capital Medical University, Beijing, 100053, China

## Abstract

Memory impairment is a typical characteristic of patients with subcortical vascular mild cognitive impairment (svMCI) or with amnestic mild cognitive impairment (aMCI). The hippocampus, which plays an important role in the consolidation of information from short-term memory to long-term memory, is a heterogeneous structure that consists of several anatomically and functionally distinct subfields. However, whether distinct hippocampal subfields are differentially and selectively affected by svMCI pathology and whether these abnormal changes in hippocampal subfields are different between svMCI and aMCI patients are largely unknown. A total of 26 svMCI patients, 26 aMCI patients and 26 healthy controls matched according to age, gender and years of education were enrolled in this study. We utilized an automated hippocampal subfield segmentation method provided by FreeSurfer to estimate the volume of several hippocampal subfields, including the cornu ammonis (CA) areas, the dentate gyrus (DG), the subiculum and the presubiculum. Compared with controls, the left subiculum and presubiculum and the right CA4/DG displayed significant atrophy in patients with svMCI. Interestingly, we also found significant differences in the volume of the right CA1 between the svMCI and aMCI groups. Taken together, our results reveal region-specific vulnerability of hippocampal subfields to svMCI pathology and identify distinct hippocampal subfield atrophy patterns between svMCI and aMCI patients.

Vascular dementia (VaD) and Alzheimer’s disease (AD) are regarded as the most common forms of dementia in the elderly. Subcortical VaD (SVaD) is a small vessel disease^1^, which constitutes approximately half of all cases of VaD^2^. SVaD is characterized by multiple lacunar infarcts and ischemic white matter hyperintensity (WMH)^3^. Similar to amnestic mild cognitive impairment (aMCI), which is thought to represent a transitional state between normal aging and AD, subcortical vascular mild cognitive impairment (svMCI) refers to a prodromal stage of SVaD^4^. The early diagnosis of svMCI may be clinically important because it is potentially reversible by modifying the vascular risk factors^5^,^6^.

Memory deficit is an important characteristic of AD and is also observed in subcortical vascular disease, although to a lesser extent compared with AD^7^,^8^. The hippocampus plays an important role in memory processing. Hippocampal atrophy has been found in patients with aMCI/AD and is widely considered a neuroimaging hallmark for the early diagnosis of AD^9^. A reduced size of the hippocampus has also been reported in svMCI and SVaD patients^10^,^11^,^12^,^13^. Moreover, the extent of hippocampal atrophy has shown a strong association with cognitive decline in subcortical ischemic vascular disease^14^,^15^.

Notably, the hippocampus is not a homogeneous structure but rather is composed of several subfields, specifically the cornu ammonis (CA) areas 1–4, the dentate gyrus (DG), the subiculum and the presubiculum^16^. These subfields have distinct histological characteristics and appear to be differentially affected by various neurodegenerative diseases^17^. An autopsy study found substantial neuronal loss in the CA1 and the subiculum in VaD patients with microvascular pathology^18^. Recently, studies employing a three-dimensional surface mapping technique have reported deformations of hippocampal shape in the lateral body (CA1) among svMCI patients, and these deformations extended to the lateral head (CA1) and the inferior body (subiculum) in SVaD patients^10^,^11^. Hippocampal subfield volumetry could provide accurate volumetric measures of all subfields within the hippocampus regardless of surface changes. Although volumetric measurement at the level of hippocampal subfields has proven to be a technological challenge due to their small size, complex shape, and considerable anatomical variability^19^, some automated segmentations of the hippocampal subfields have recently been developed and established to be effective^20^,^21^. The method developed by Van Leemput *et al.*^21^, which has been implemented in the public software FreeSurfer, was based on a Bayesian modeling approach to label the hippocampal subfields. An analysis of hippocampal subfield volumes using FreeSurfer has been successfully applied in many neuropsychiatric diseases^22^,^23^,^24^,^25^. However, whether the volumes of various hippocampal subfields in svMCI patients display distinct changes (i.e., heterogeneity) relative to their respective volumes in normal controls is largely unknown.

SVaD/svMCI may not be easily clinically discriminated from AD/aMCI because the clinical symptoms and the neuroimaging features of these two types of dementia overlap^26^,^27^. For example, WMH disruptions on MR images are required to diagnose SVaD/svMCI^28^, although this abnormality has also been observed in AD patients^29^. Additionally, hippocampal atrophy, an important biomarker of AD, has also been detected in SVaD patients^12^,^13^. However, the etiologies of svMCI and aMCI are different^27^, as svMCI is caused by small vessel abnormalities^1^ and aMCI results from neurodegenerative changes^30^. The distinct pathophysiological mechanism underlying each type of dementia could produce different abnormal patterns of the hippocampal subfields, and such evidence would be helpful for discriminating between these two types of dementia at an early stage.

The purposes of the present study were to 1) investigate whether hippocampal subfields in svMCI patients display different patterns of atrophy from those in normally aging subjects and 2) compare the patterns of hippocampal atrophy between svMCI and aMCI patients. We first performed segmentation of the hippocampal subfields using a fully automated method provided by FreeSurfer^21^; then, we statistically compared the subfield volumes between the groups. As mentioned above, the hippocampal subfields have distinct histological characteristics and appear to be differentially affected by various neurodegenerative diseases^17^; thus, we expected to observe different patterns of atrophy in the hippocampal subfields of svMCI patients compared with normal control (NC) subjects and with aMCI patients.

## Results

### Demographic data

Table 1 shows the demographic data of the controls, the patients with svMCI, and the patients with aMCI. The three groups were well matched in terms of sex. There was no significant difference in age (F(2,75) = 0.269, *p* = 0.765) or years of education (F(2,75) = 0.513, *p* = 0.601) between the groups. Additionally, the number of vascular risk factors was significantly fewer (*p* < 0.005 based on Bonferroni *post hoc* analysis) in both the NC and aMCI groups compared to the svMCI group. Furthermore, significant differences in the occurrence of arterial hypertension (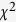 = 9.199, *p* = 0.010), diabetes mellitus (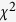 = 10.123, *p* = 0.006), and heart disease (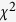 = 14.679, *p* = 0.001) were found between the three study groups. *Post hoc* tests revealed that the NC group exhibited lower rates of arterial hypertension and heart disease than the svMCI group and that the aMCI group displayed lower rates of diabetes mellitus and heart disease than the svMCI group. Moreover, as determined by one-way analysis of variance (ANOVA), between-group differences in the Mini Mental Status Examination (MMSE) (F(2,75) = 8.473, *p* < 0.001), Clock Drawing Test (CDT) (F(2,75) = 5.704, *p* = 0.005), Auditory Verbal Learning Test (AVLT)-immediate recall (F(2,75) = 28.551, *p* < 0.001), AVLT-delayed recall (F(2,75) = 22.642, *p* < 0.001) and AVLT-recognition scores (F(2,75) = 7.812, *p  *= 0.001) were observed. *Post hoc* analysis revealed that all of these neuropsychological test scores were significantly lower in both the svMCI and aMCI groups than in the NC group (*p* < 0.05). However, these test scores were not significantly different between the svMCI and aMCI groups (*p* > 0.05).

### Comparisons of hippocampal subfield volumes

**Figure 1:**
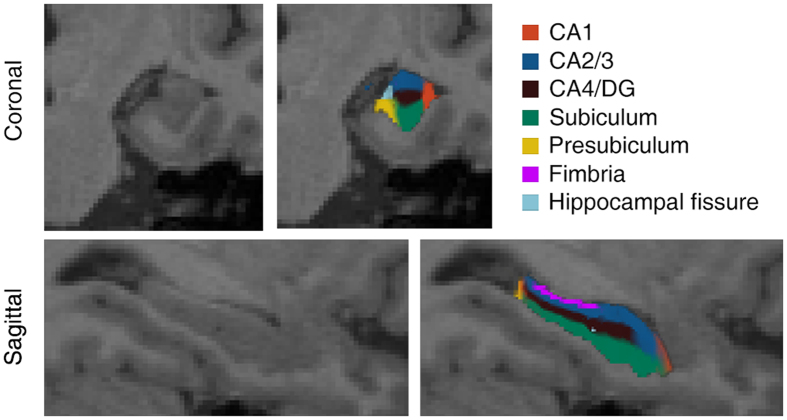
Hippocampal subfield segmentation. A coronal view (top) and a sagittal view (bottom) are shown.

**Figure 2:**
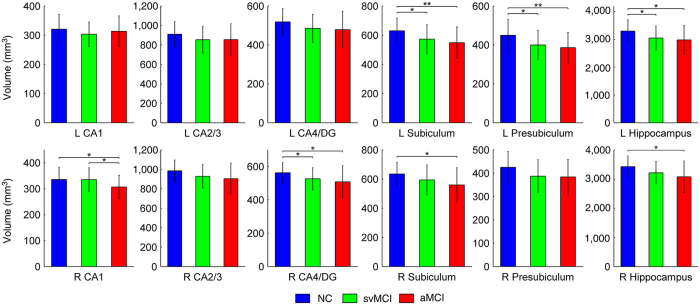
Between-group comparisons of hippocampal volumetric measurements. ANOVA followed by Bonferroni *post hoc* analysis was performed (*p < 0.05; **p < 0.01). NC, normal controls; svMCI, subcortical vascular mild cognitive impairment; aMCI, amnestic mild cognitive impairment; CA, cornu ammonis; DG, dentate gyrus.

Figure 1 illustrates the subfield segmentation results for one of the participants in the current study. The statistical results of the hippocampal subfields and the total hippocampal volumes are shown in Table 2. The volume of the hippocampus in the svMCI patients tended to be intermediate between the aMCI patients and the NC subjects. The volumetric differences between the groups are illustrated in Fig. 2. Based on *post hoc* analysis, we found a significant decrease in the volume of the left hippocampus in aMCI patients (*p* = 0.020) as well as svMCI patients (*p* = 0.043) and in volume of the right hippocampus of aMCI patients (*p* = 0.007) compared with NC subjects. However, no significant difference in the bilateral hippocampal volume was detected between the aMCI and svMCI groups (*p* > 0.05). In the subfield volumetric analyses, we found significant reductions in the volume of the left subiculum (*p* = 0.036), presubiculum (*p* = 0.024) and the right CA4/DG (*p* = 0.038) in the svMCI group compared with the NC group. However, we observed additional hippocampal subfields displaying further volumetric atrophy in the aMCI patients than in the NC subjects: the bilateral subiculum (left: *p = *0.005; right: *p* = 0.012), the left presubiculum (*p = *0.009), the right CA1 (*p* = 0.030), and the CA4/DG (*p* = 0.017). Interestingly, we also detected a significant difference in the volume of the right CA1 between the svMCI and aMCI groups (*p* = 0.033).

## Discussion

In this study, we found that hippocampal subfields were differentially and selectively affected by svMCI pathology. Specifically, svMCI patients showed significantly reduced hippocampal subfield volumes in the left subiculum, the presubiculum and the right CA4/DG relative to healthy control subjects. Additionally, different hippocampal atrophy patterns were observed between svMCI patients and aMCI patients; the aMCI patients showed more extensive hippocampal subfield atrophy than the svMCI patients.

Hippocampal atrophy in svMCI/SVaD patients had been found in several previous neuroimaging studies^11^,^12^,^14^. Cortical hypometabolism and hypoperfusion, which occur in SVaD patients, could contribute to hippocampal and cortical atrophy^31^. Furthermore, experiments using animal models of ischemia demonstrated that reducing cerebral blood flow causes memory and behavioral impairments and neuronal loss in the hippocampus, the striatum, and the cerebral cortex^31^. Moreover, the hippocampus was demonstrated to be one of the most vulnerable structures to brain ischemia; thus, hippocampal volume loss may be attributed to delayed neuronal death caused by chronic ischemia^32^. Additionally, hippocampal damage might indirectly result from disrupted connections of the hippocampus with cortical areas, and these disruptions could lead to secondary degeneration of hippocampal neurons^14^.

We observed a volumetric reduction in the left subiculum in svMCI patients. In line with this finding, an autopsy study of patients with SVaD reported significant atrophy and neuronal loss in grey matter regions of the subiculum^18^. Similarly, a recent study reported that the shape of the subiculum was deformed in patients with SVaD based on a surface mapping method^10^. Atrophy of the subiculum in svMCI/SVaD could be explained by increased glucocorticoid density^33^ compared with other subregions^34^,^35^ because a previous animal study demonstrated that glucocorticoid signaling can exacerbate ischemic injury (one type of vascular pathology) to neurons in the rat brain^36^. Additionally, VaD patients showed altered activity in the hypothalamic-pituitary-adrenal axis^37^. We speculate that this alteration could be caused by a reduction in the volume of the subiculum because the ventral subiculum is involved in inhibiting hypothalamic-pituitary-adrenal axis activity^38^. Moreover, the dorsal subiculum plays a role in information processing functions, including memory functions^38^, and loss of volume in the subiculum may account for memory dysfunction in svMCI patients.

Another finding was a loss of volume in the left presubiculum in svMCI patients. The presubiculum serves as a relay station in the hippocampal neuronal circuitry^39^ and receives input from several cortical areas. Therefore, pathology affecting the presubiculum in svMCI patients disturbs the flow of information through hippocampal neural circuits, resulting in disrupted connections of the hippocampus with multiple cortical regions^40^. As the entire hippocampal neuronal circuit plays a crucial role in maintaining memory functions, malfunction of the presubiculum subregion may underlie memory impairment and associated symptoms in patients with svMCI.

Significant atrophy of the right CA4/DG subfield was also observed in svMCI patients. A previous study of the gerbil hippocampus showed that the CA4 region was extremely susceptible to ischemic insults but that the DG region showed ischemic damage only after a long-term ischemic insult^41^. Additionally, the subgranular zone of the DG is among the few regions of the adult mammalian brain in which newborn granule neurons are generated throughout life^42^,^43^. Animal experiments demonstrated that although ischemia can stimulate neurogenesis^44^, the self-repair potential of the adult mammalian brain is insufficient because the majority of newly generated cells die over time in an ischemic microenvironment^45^,^46^. This evidence may explain the DG abnormality found in svMCI patients in this study.

We did not observe any significant difference in the CA1 subregion volume between the svMCI group and the control group, and this observation was compatible with the finding in a previous study that the neuronal count in the CA1 subregion was unchanged in ischemic VaD patients compared to control subjects^47^. However, the CA1 subfield of the gerbil hippocampus appears to be vulnerable to anoxic-ischemic insults^41^. Significant atrophy and neuronal loss of the CA1 region were found in an autopsy study of patients with microvascular pathology^18^. Moreover, a recent study revealed a in the shape of the lateral body (CA1) region in svMCI patients based on a 3D surface mapping technique^11^. Notably, those results are not directly comparable to our results, as the shape-based method indirectly reflects the localized volume loss in the hippocampus by detecting the deformed surface boundaries of the hippocampus. Additionally, the definition of each hippocampal subfield might be different, as suggested by Hanseeuw *et al*^48^. Specifically, according to segmentation using FreeSurfer, the CA1 area is a small region relative to other hippocampal subfields (as shown in Table 2), whereas the CA1 area is one of the largest subfields according to anatomic atlases^16^. Thus, the CA1 results in our study should be interpreted with caution.

We also found that hippocampal atrophy in the svMCI group was more lateralized towards the left hemisphere (i.e., significant volume reductions were found in the entire hippocampus, particularly in the left subiculum and presubiculum and in the right CA4/DG). There exists hippocampal asymmetry in healthy adults, in which the left hippocampus has a smaller volume than the right hippocampus^49^. We speculated that the left hippocampus was more vulnerable to vascular pathology than the right hippocampus due to its naturally smaller volume. Comparably, a previous study reported that patients with svMCI had a smaller shape in the left anterior hippocampus and that patients with moderate-to-severe subcortical vascular cognitive impairment had a smaller shape in the bilateral anterior hippocampus^50^. Their results may indicate that the left hippocampus was the first region affected by subcortical vascular pathology, followed by atrophy in the right hippocampus with disease progression.

Our results indicated that patients with svMCI had less hippocampal atrophy than patients with aMCI, although the two groups exhibited similar cognitive impairment (no significant difference in MMSE scores between the groups). These findings are comparable with those of previous studies, which reported that hippocampal atrophy in SVaD was milder than that in AD^10^,^12^. Specifically, we found a significantly smaller right CA1 in aMCI patients than in svMCI patients. Significant atrophy of the subiculum was detected exclusively in the left hippocampus of svMCI patients but was observed bilaterally in aMCI patients. The CA1 and subicular subregions were demonstrated to be the earliest regions affected by neurofibrillary tangle-related lesion development in AD pathology^51^. Additionally, the authors of a recent study speculated that the subiculum is more vulnerable to AD pathology than vascular pathology^10^. Our findings are also supported by a previous pathology study that reported less atrophy of the CA1 and the subiculum in SVaD patients than in AD patients^18^. The different hippocampal atrophy patterns may be associated with the distinct severities of memory disturbances between patients with svMCI and aMCI. Further studies exploring the relationship between differences in hippocampal subfield volumes and the distinct pathophysiological mechanisms underlying each type of dementia (microvascular vs. neurodegenerative) are needed.

Several issues related to this study must be mentioned. First, one critical limitation of this study is that we could not completely discriminate between mixed AD and vascular pathology according to our inclusion criterion (i.e., patients with clinically diagnosed svMCI may harbor AD pathology). The recently developed ^11^C-Pittsburg compound-B positron emission tomography technique may partially exclude those harboring AD pathology^52^. This technique could be used in our future research. Second, the definitions of the hippocampal subfield boundaries obtained using FreeSurfer may be different from those obtained by other research groups. Thus, caution is needed in comparing our results with the findings of other studies. However, the hippocampal subfield segmentation method provided by FreeSurfer was validated against manual segmentation and achieved reasonable Dice index values (approximately 0.7) for all large hippocampal subfields^21^. The reproducibility of this method was confirmed based on the similar hippocampal subfield atrophy patterns among various studies of patients with aMCI^48^ and AD^24^. Finally, svMCI can be classified into various subtypes according to its progressive clinical trajectory (i.e., some svMCI patients progress to SVaD, but others do not). It would be interesting to longitudinally examine the distinct changes in hippocampal subfield volumes in svMCI subgroups in future studies.

In conclusion, we found hippocampal subfield volume reductions in the left subiculum and presubiculum and in the right CA4/DG in patients with svMCI compared to control subjects. Additionally, we observed that hippocampal atrophy was less severe in patients with svMCI than in those with aMCI. These findings help to increase our understanding of svMCI pathology and to more effectively distinguish between svMCI and aMCI in patients.

## Methods

### Participants

Patients with svMCI and aMCI were recruited from September 2009 to October 2011 through the memory clinic of the neurology department of Xuanwu Hospital, Capital Medical University, Beijing, China. All patients were diagnosed by two experienced neurologists. The patients were diagnosed with svMCI using the Petersen criteria^53^ with the following modifications that were previously described^54^: i) subjective cognitive complaint reported by the participant, preferably confirmed by his/her caregiver; ii) objective cognitive decline below 1.5 SD of the age- and education-adjusted norms on neuropsychological tests; iii) normal or near-normal performance of general cognitive function as assessed by the MMSE (MMSE score ≥24 for middle school-educated, ≥20 for primary school-educated, and ≥17 for illiterate participants)^55^ and no or minimal impairments in activities of daily living (ADL) as assessed by both the ADL scale (ADL score <26) and an interview with a clinician; iv) a CDR score = 0.5; v) not demented according to the Diagnostic and Statistical Manual of Mental Disorders, 4th edition, revised (DSM-IV) criteria; vi) at least one region displaying moderate to severe WMH (Wahlund rating scale score^56^ ≥2) and/or multiple lacunar infarcts in the periventricular and deep white matter (Wahlund rating scale score ≥2; diameter <15 mm). A lacuna was defined as a small lesion with high signal intensity on T2-weighted images or a perilesional halo on FLAIR images; and vii) focal neurological symptoms or signs observed in a general or neurological examination or reported by the participant or his/her caregiver. Additionally, all aMCI patients fulfilled the Petersen criteria^53^ with the following modifications: i) subjective memory complaint reported by the participant, preferably confirmed by his/her caregiver; ii) objective memory decline below 1.5 SD of the age- and education-adjusted norms on memory-related neuropsychological tests; iii) no or very mild white matter changes or brain infarcts on T2/FLAIR images; and iv) the same diagnostic criteria as iii)-v) in the above list for svMCI. Patients were excluded if they exhibited any of the following clinical characteristics: i) severe depressive symptoms based on a Hamilton Depression Rating Scale score^57^ >24; ii) cognitive impairments caused by psychiatric disease, systemic disease (e.g., thyroid dysfunction, severe anemia, syphilis or HIV), non-MCI neurological disorders, or alcohol or drug abuse; or iii) visual abnormalities, severe aphasia or motor disorders that would render neuropsychological testing infeasible. According to the inclusion and exclusion criteria, 26 patients were diagnosed with svMCI, and 37 patients were diagnosed with aMCI. Fifty-five NC subjects were recruited from the local community using advertisements. Those subjects were screened using the Structured Interview for DSM-IV, Non-Patient Edition, to confirm that they had no history of any neurological or psychiatric disorders.

Then, we randomly selected 26 aMCI patients and 26 NC subjects who matched the svMCI patients according to age, gender and years of education. All participants underwent a standardized clinical evaluation protocol, which included general and neurological examinations, a global cognitive functioning test (i.e., CDR and MMSE) and other cognitive assessments (i.e., ADL, CDT and AVLT). We analyzed the patients’ clinical records to record the number of vascular risk factors (i.e., arterial hypertension, diabetes mellitus, hypercholesterolemia and heart disease) for each participant. The detailed demographic characteristics of the participants are shown in Table 1.

This study was approved by the medical research ethics committee and the institutional review board of Xuanwu Hospital, Capital Medical University, Beijing, China. The study was conducted in accordance with the approved guidelines. Written informed consent was obtained from all participants.

### Data acquisition

MR imaging was performed using a 3.0 T Siemens scanner at Xuanwu Hospital, Capital Medical University. Structural images were acquired using a sagittal magnetization-prepared rapid gradient echo (MP-RAGE) three-dimensional T1-weighted imaging sequence: repetition time (TR) = 1900 ms; echo time (TE) = 2.2 ms; inversion time = 900 ms; flip angle = 9°; field of view (FOV) = 256 mm × 256 mm; matrix = 256 × 256; 176 slices; and slice thickness = 1.0 mm.

### Image processing

Structural MR images were processed using the FreeSurfer image analysis suite, which can be freely downloaded from a web site (version 5.3.0, http://freesurfer.net/). First, the entire hippocampal formation was segmented using the routine volumetric FreeSurfer pipeline. Briefly, T1-weighted MR images were corrected for within-subject head motion; then, non-brain tissues were removed using a hybrid watershed/surface deformation algorithm^58^. The resulting images were further affine registered to the Talairach space. Subsequently, segmentation of the subcortical and cortical structures (including the hippocampus) was conducted using a probabilistic brain atlas^59^. The estimated total intracranial volume (eTIV) of each subject was also calculated using the standard FreeSurfer processing pipeline by exploiting the relationship between the intracranial volume and the linear transformation to the atlas template^60^. The eTIV was used to correct for individual differences in head size in the subsequent statistical analysis.

Automated segmentation of hippocampal subfields was performed using a built-in module of FreeSurfer, in which a Bayesian statistical model with Markov random field priors was used to estimate the label of each subfield^21^. This method has been successfully applied to detect hippocampal abnormalities in specific subfields in many neuropsychiatric diseases^22^,^23^,^24^,^25^. A bounding box containing the hippocampus that was upsampled to a 0.5 mm isotropic resolution was applied to this module. This approach relied on a tetrahedral mesh-based probabilistic atlas of the hippocampal formation, which was constructed from the manual delineation of the right hippocampus based on ultra-high-resolution T1-weighted scans (0.38 × 0.38 × 0.8 mm^3^) of 10 normal subjects. By maximizing the posterior probability of a segmentation, the left and right hippocampi were automatically segmented into seven subfields: CA1, CA2/3, CA4/DG, subiculum, presubiculum, fimbria and hippocampal fissure. The hippocampal subfield segmentation results are illustrated in Fig. 1. The entire hippocampal volume was defined as the sum of the volume of all hippocampal subfields. We disregarded the fimbria and the hippocampal fissure in the subsequent analysis because the segmentation of these two small subfields has been shown to be inaccurate (Dice index values of 0.51 and 0.53, respectively), as reported by Kühn, S. *et al*^23^.

### Statistical analysis

Statistical analysis was conducted using Statistical Package for Social Sciences (SPSS, version 20.0) software. All statistical tests were two-tailed. Continuous demographic variables were evaluated via ANOVA. Statistically significant differences based on ANOVA (*p* < 0.05) were further explored using Bonferroni *post hoc* analysis. Moreover, categorical demographic variables were evaluated using the Chi-square test. Additionally, correlation analyses were performed to examine the relationships between hippocampal subfield volumes and eTIV/age. No significant correlations were found between these parameters. Therefore, ANOVA was performed to evaluate the differences in the entire hippocampal volume and in hippocampal subfield volumes between groups, considering group (i.e., svMCI, aMCI and NC) as a fixed factor. If a significant effect of group was found (*p* < 0.05), *post hoc* analysis using Bonferroni’s method was implemented for pairwise comparisons. ANOVA was separately performed for the left and right hemispheres.

## Additional Information

**How to cite this article**: Li, X. *et al.* Hippocampal subfield volumetry in patients with subcortical vascular mild cognitive impairment. *Sci. Rep.* **6**, 20873; doi: 10.1038/srep20873 (2016).

## Figures and Tables

**Table 1:** Demographic characteristics of the participants (means ± standard deviation, (range)).

	NC (n = 26)	svMCI (n = 26)	aMCI (n = 26)
Gender (M/F)	11/15	11/15	align="center">11/15
Age (years)	65.4 ± 8.1 (43–79)	67.0 ± 9.9 (46–79)	65.7 ± 7.1 (52–79)
Years of education	10.3 ± 4.1 (0–17)	10.3 ± 4.2 (0–18)	9.8 ± 5.0 (0–21)
Number of vascular risk factors	0.9 ± 1.2 (0–4)	2.0 ± 0.9 (0–4)^*^	0.8 ± 0.9 (0–3)^+^
Arterial hypertension, *N* (%)	8 (26)	18 (69)^*^	11 (42)
Diabetes mellitus, *N* (%)	7 (27)	13 (50)	3 (12)^+^
Hypercholesterolemia, *N* (%)	5 (19)	6 (23)	6 (23)
Heart disease, *N* (%)	3 (12)	11 (42)^*^	1 (4)^+^
CDR	0	0.5	0.5
MMSE	27.9 ± 2.4 (20–30)	25.9 ± 2.4 (21–30)^*^	24.9 ± 3.3 (18–30)^*^
CDT	2.8 ± 0.5 (1–3)	2.3 ± 0.8 (1–3)^*^	2.2 ± 0.8 (0–3)^*^
AVLT-immediate recall	8.9 ± 2.0 (5.0–14.7)	5.4 ± 2.1 (3.3–9.7)^*^	5.7 ± 1.4 (3.0–8.0)^*^
AVLT-delayed recall	9.7 ± 2.6 (5–15)	5.7 ± 3.3 (0–12)^*^	4.8 ± 2.6 (0–10)^*^
AVLT-recognition	11.9 ± 2.2 (7–15)	9.5 ± 3.3 (4–14)^*^	8.4 ± 3.8 (1–14)^*^

^*,+^ANOVA followed by Bonferroni *post hoc* analysis for continuous variables or the Chi-square test for categorical variables: ^*^p < 0.05 between NC and svMCI or aMCI; ^+^*p* < 0.05 between svMCI and aMCI. n = number of subjects; NC, normal control group; svMCI, subcortical vascular mild cognitive impairment group; aMCI, amnestic mild cognitive impairment group; CDR, Clinical Dementia Rating; MMSE, Mini Mental Status Examination; CDT, Clock Drawing Test; AVLT, Auditory Verbal Learning Test.

**Table 2:** Volumes of the hippocampal subfields and the total hippocampus (means ± standard deviation, mm^3^).

	Left			Right		
	NC	svMCI	aMCI	NC	svMCI	aMCI
CA1	321 ± 51	304 ± 41	314 ± 52	336 ± 46	335 ± 45	307 ± 45^*,+^
CA2/3	911 ± 130	855 ± 138	855 ± 163	986 ± 110	929 ± 121	904 ± 160
CA4/DG	519 ± 68	486 ± 72	479 ± 94	635 ± 80	594 ± 102^*^	559 ± 117^*^
Subiculum	630 ± 87	573 ± 98^*^	549 ± 108^*^	563 ± 60	527 ± 66	508 ± 96^*^
Presubiculum	450 ± 81	400 ± 74^*^	387 ± 79^*^	425 ± 68	387 ± 71	384 ± 75
Hippocampus	3300 ± 407	3050 ± 439^*^	2983 ± 527^*^	3428 ± 365	3217 ± 389	3079 ± 539^*^

^*,+^ANOVA followed by Bonferroni *post hoc* analysis: ^*^p < 0.05 between NC and svMCI or aMCI; ^+^*p* < 0.05 between svMCI and aMCI. NC, normal control group; svMCI, subcortical vascular mild cognitive impairment group; aMCI, amnestic mild cognitive impairment group; CA, cornu ammonis; DG, dentate gyrus.
